# IL-1β contributes to neurological disability in NMOSD AQP4 + Patients

**DOI:** 10.1007/s10072-025-08526-8

**Published:** 2025-10-08

**Authors:** Antonio Bruno, Angela Borrelli, Gianluca Lauritano, Sonia Di Lemme, Veronica Di Caprio, Roberta Fantozzi, Ettore Dolcetti, Federica Azzolini, Luana Gilio, Giovanni Galifi, Roberto Furlan, Annamaria Finardi, Francesca De Vito, Alessandra Musella, Georgia Mandolesi, Mario Stampanoni Bassi, Diego Centonze, Fabio Buttari

**Affiliations:** 1 IRCCS Neuromed, Via Atinense, 18, Pozzilli (IS), 86077 Italy; 2 Faculty of Psychology, International Telematic University Uninettuno, Rome, Italy; 3 Clinical Neuroimmunology Unit, Institute of Experimental Neurology (INSpe), Division of Neuroscience, San Raffaele Scientific Institute, Milan, Italy; 4 Synaptic Immunopathology Lab, IRCCS San Raffaele Roma, Roma, Italy; 5 Department of Human Sciences and Quality of Life Promotion, University of Rome San Raffaele, Rome, Italy; 6 Department of Systems Medicine, University of Rome Tor Vergata, Rome, Italy

**Keywords:** NMOSD, Cytokines, Disability, IL-1β, Aquaporin-4, Neuroinflammation

## Abstract

**Background:**

Neuromyelitis Optica Spectrum Disorder (NMOSD) is a central nervous system inflammatory disease that causes severe disability. Differently from relapsing-remitting multiple sclerosis (RRMS) a group of patients present aquaporin-4 (AQP4) antibodies in the serum. Although its pathogenesis is unclear, cytokine profiles may impact disease activity and severity.

**Objective:**

To analyze cerebrospinal fluid (CSF) cytokine levels in NMOSD AQP4 + and their relationship with neurological disability, comparing findings with RRMS patients and non-inflammatory controls (NIC).

**Methods:**

Sixty-four participants were recruited: 11 NMOSD AQP4+, 29 RRMS, and 24 NIC. CSF cytokine levels were measured using a multiplex assay. Group comparisons were performed with Kruskal-Wallis and Mann-Whitney U tests, while linear regression models evaluated the association between cytokine levels and Expanded Disability Status Scale (EDSS) scores.

**Results:**

NMOSD AQP4 + patients displayed significantly higher CSF levels of IL-1β (*p* = 0.040), TNF-α (*p* < 0.001), G-CSF (*p* = 0.003), Eotaxin (*p* = 0.008), and MIP-1α (*p* = 0.005) compared to RRMS and NIC. Moreover, IL-1β CSF levels were positively associated with disability at the time of lumbar puncture (β = 22.24, SE = 7.73, *p* = 0.018), a relationship that remained significant after adjusting for age (β = 18.96, SE = 6.33, *p* = 0.040).

**Conclusions:**

Expression of proinflammatory cytokines may differ between NMOSD and RRMS. Elevated IL-1β levels in NMOSD AQP4 + patients are associated with neurological disability, suggesting a potential role as a biomarker of disease severity. Further studies are needed to confirm these findings and evaluate the therapeutic potential of targeting IL-1β in NMOSD.

**Supplementary Information:**

The online version contains supplementary material available at 10.1007/s10072-025-08526-8.

## Highlights

NMOSD AQP4 + patients show distinct cytokine profiles compared to RRMS and NIC.

Elevated IL-1β levels are associated with disability in NMOSD AQP4 + patients.

IL-1β could serve as a potential biomarker and therapeutic target in NMOSD.

**Supplementary Information:**

The online version contains supplementary material available at 10.1007/s10072-025-08526-8.

## Introduction

Neuromyelitis Optica Spectrum Disorder (NMOSD) is a group of severe inflammatory demyelinating disorders of the central nervous system (CNS) characterized by debilitating attacks of bilateral optic neuritis and longitudinally extensive transverse myelitis [1]. A significant subset of NMOSD cases, also referred to as Devic’s disease, is associated with antibodies targeting aquaporin-4 (AQP4), a water channel protein predominantly expressed in astrocytes [2]. Although both NMOSD AQP4 + and relapsing-remitting multiple sclerosis (RRMS) are autoimmune inflammatory diseases, NMOSD AQP4 + is distinguished by a more severe clinical course, with higher symptom intensity and greater residual disability [1]. Several studies have demonstrated significantly elevated cytokine levels in the cerebrospinal fluid (CSF) of NMOSD AQP4 + patients, including IL-6, IL-17, IL-1β, IL-12, interferon-gamma (IFN-γ), B-cell activating factor (BAFF), CXCL13, and tumor necrosis factor-alpha (TNF-α), compared to RRMS and healthy controls [3–7]. Among these, IL-6 has received particular attention, as evidenced by the efficacy of Satralizumab, an IL-6 receptor antibody, in reducing the frequency of NMOSD AQP4 + relapses [8]. Given the recurrent nature of NMOSD and its potential to cause severe, permanent neurological disability, current treatments primarily aim to reduce relapse frequency and severity [2]. Further elucidation of the inflammatory mechanisms underlying the greater severity and disability in NMOSD AQP4 + compared to RRMS is essential for developing targeted therapeutic strategies and improving patient outcomes. In this study, we analyzed a cohort of patients with NMOSD AQP4+, RRMS, and a population of non-inflammatory controls (NIC) to evaluate differences in CSF inflammatory profiles and their potential impact on disability.

## Materials and methods

### Patients enrollment

In this study, we enrolled a total of 64 patients divided into three groups: NMOSD AQP4+ (*n* = 11), RRMS (*n* = 29), and non-inflammatory controls (NIC, *n* = 24) at the time of diagnosis. Participants were recruited from the neurological clinic of the Neuromed Research Institute in Pozzilli, Italy, between 2017 and 2019. At enrollment, clinical parameters, including age, sex, and Expanded Disability Status Scale (EDSS) scores, were recorded for all participants. The diagnosis of NMOSD AQP4 + and MS was established based on clinical evaluation, laboratory tests, and MRI findings. NMOSD AQP4 + patients were included if they had a confirmed diagnosis according to established diagnostic criteria, including the presence of AQP4-IgG antibodies. RRMS patients were diagnosed according to the McDonald diagnostic criteria. Non-inflammatory controls (NIC) were selected from individuals without a history of inflammatory neurological disorders and were matched for age, sex, and EDSS with the patient groups. Exclusion criteria included the presence of other neurological conditions, recent immunosuppressive therapy, a history of systemic inflammatory diseases, or the inability to provide informed consent.

The Ethics Committee of the Neuromed Research Institute approved the study in accordance with the Declaration of Helsinki (cod. 06–17). All participants provided written informed consent prior to enrollment. At the time of diagnosis, all patients underwent comprehensive clinical evaluations, including brain and spinal MRI and lumbar puncture (LP). Clinical characteristics recorded included age, sex, Expanded Disability Status Scale (EDSS) scores, and disease duration. Disease duration was defined as the time elapsed between the first clinical episode indicative of NMOSD AQP4 + or MS and the confirmation of diagnosis. Relapse distance was defined as the interval from the LP to the last clinical relapse for both NMOSD AQP4 + and RRMS patients.

### CSF Collection and Analysis

In all RR-MS patients CSF concentrations of inflammatory cytokines were analyzed. CSF was collected at the time of diagnosis, during hospitalization, by LP. No corticosteroids were administered before LP. Disease modifying therapies were initiated after the confirmed diagnosis when indicated. CSF was stored at −80° C and then analyzed using a Bio-Plex multiplex cytokine assay (Bio-Rad Laboratories, Hercules, CA, USA). CSF cytokines levels were determined according to a standard curve generated for the specific target and expressed as picograms/milliliter (pg/mL). Samples were analyzed in triplicate. The CSF cytokines analyzed included IL-1β, IL-2, IL-4, IL-5, IL-6, IL-7, IL-8, IL-9, IL-10, IL-12, IL-13, IL-15, IL-17 tumor necrosis factor α (TNF-α), IFN-γ, macrophage inflammatory protein 1α (MIP-1α), macrophage inflammatory protein 1β (MIP-1β), monocyte chemoattractant protein 1 (MCP-1), granulocyte colony-stimulating factor (G-CSF), granulocyte-monocyte colony stimulating factor (GM-CSF), interleukin-1 receptor antagonist (IL-1ra), eotaxin, fibroblast growing factor (FGF), IFN-γ induced protein 10 (IP-10), platelet-derived growth factor (PDGF), regulated upon activation, normal T cell expressed and secreted (RANTES), vascular endothelial growth factor (VEGF).

### MRI

All patients underwent magnetic resonance imaging (MRI) of the brain and spinal cord using a 1.5T MRI scanner. The imaging protocol included the following sequences: dual-echo proton density, fluid-attenuated inversion recovery (FLAIR), T1-weighted spin-echo (SE), T2-weighted fast SE, and contrast-enhanced T1-weighted SE. Contrast enhancement was achieved by administering intravenous gadolinium (Gd) at a dosage of 0.2 mL/kg. The presence of gadolinium-enhancing (Gd+) lesions at the time of diagnosis was defined as the detection of Gd + lesions in the brain and spinal cord during hospitalization.

### Statistical analysis

The Shapiro-Wilk test was utilized to evaluate the normality of continuous variable distributions. Data are presented as mean (standard deviation, SD) or median (interquartile range, IQR), depending on the normality of the data. Categorical variables are expressed as absolute counts (n) and relative frequencies (%). To explore associations between categorical variables, we employed the Chi-square test or, when appropriate, Fisher’s exact test. Differences in continuous variables were assessed using the nonparametric Mann-Whitney U test for comparisons between groups. Additionally, the Kruskal-Wallis test was used to evaluate differences in cytokine levels among three groups (NIC, NMOSD AQP4+, and RRMS). Post-hoc analyses were conducted using the Mann-Whitney U test to determine specific group differences where significant variations were identified. A p-value of < 0.05 was considered statistically significant. All statistical analyses were conducted using IBM SPSS Statistics for Windows (IBM Corp., Armonk, NY, USA).

## Results

### Clinical and demographical data

Table 1 Summarizes the clinical and demographic characteristics of patients with NMOSD AQP4 + and RRMS at the moment of LP. No significant differences were found in clinical characteristics between NMOSD and RRMS. There were no significant differences in age (Mann-Whitney U test, *p* = 0.517) or sex distribution (Fisher’s exact test, *p* = 0.782) between NMOSD AQP4 + and NIC. The clinical presentation of RRMS patients was as follows: optic neuritis (NORB) (*n* = 2, 6.9%), medullary involvement (*n* = 15, 51.7%), cerebral symptoms (*n* = 6, 20.8%), cerebellar symptoms (*n* = 3, 10.3%), and brainstem symptoms (*n* = 3, 10.3%). The diagnoses for NIC included headache (*n* = 4, 16.7%), vascular leukoencephalopathy (*n* = 6, 25%), idiopathic axonal neuropathy (*n* = 10, 41.7%), pseudotumor cerebri (*n* = 3, 12.5%), and idiopathic normal pressure hydrocephalus (*n* = 1, 4.2%).

**Table 1:** Demographic and Clinical DataDemographic and Clinical Data

Characteristic		NMOSD-AQP4+ (*n* = 11)	RRMS (*n* = 29)	*p*-value
Sex, F	N (%)	10 (90.9%)	24 (82.8%)	1.0
Age, years	Mean, (SD)	57.01 (13.09)	52.96 (4.58)	0.4
Disease duration, months	Median (IQR)	9.00 (6.25-74.00)	24.00 (6.13–60.40)	0.67
Relapse Distance, months	Median (IQR)	6.00 (2.50-7.00)	7.80 (5.30-12.27)	0.06
Gd + at MRI	N (%)	4.0 (36.4%)	4.0 (13.8%)	0.18
EDSS	Median (IQR)	3.00 (2.50–6.75)	2.50 (1.50–3.50)	0.054

Abbreviations: *F* female, *NMOSD* neuromyelitis optica spectrum disorder, *AQP4* aquaporin-4, *RRMS* relapsing-remitting multiple sclerosis, *EDSS* expanded disability status scale, *OCB* oligoclonal bands, *N* number, *SD* standard deviation, *IQR* interquartile range. Missing data: EDSS 1 year: 3

Continuous variables are presented as Median (Interquartile Range, IQR) or Mean (Standard Deviation, SD), while categoricalvariables are presented as absolute numbers (N) and percentages (%).

Statistical significance (p < 0.05) was determined using the nonparametric Mann–Whitney U test for continuous variables and Fisher’s exact test for categorical variables.

### Differences in CSF cytokines levels

**Fig. 1:**
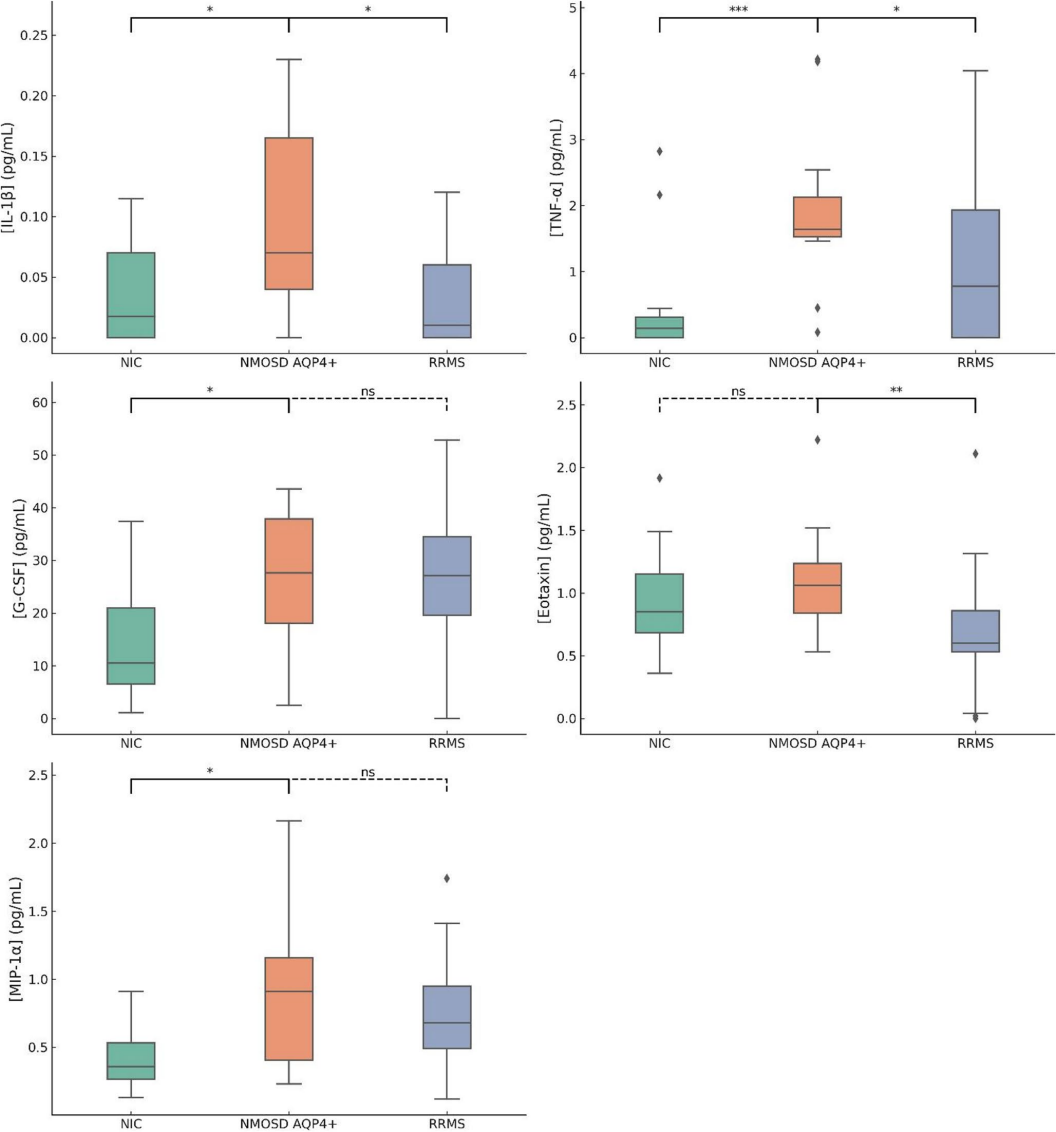
Differences in CSF Cytokines levels. Kruskal-Wallis tests were performed to evaluate the differences in cytokine levels among the three groups: Non-Inflammatory Controls (NIC), NMOSD AQP4+, and RRMS. Post-hoc Mann-Whitney U tests were conducted to compare cytokine levels between individual groups. Significant variations were observed for IL-1β (*p* = 0.040), G-CSF (*p* = 0.003), TNF-α (*p* < 0.001), Eotaxin (*p* = 0.008), and MIP-1α (*p* = 0.005). An asterisk (*) denotes statistical significance at post-hoc analysis (*p* < 0.05). Abbreviations: NIS: Non-Inflammatory Controls; NMOSD: Neuromyelitis optica spectrum disorder; AQP4: Aquaporin-4; RRMS: Relapsing-Remitting Multiple Sclerosis; CSF: cerebrospinal fluid; IL: Interleukin; G-CSF: granulocyte-colony stimulating factor; TNF-α: tumor necrosis factor-alpha; MIP-1α: Macrophage Inflammatory Protein-1 alfa; Eotaxin. Missing data: eotaxin: 2 RRMS; MIP-1α: 2 RRMS

Kruskal-Wallis tests were performed to evaluate the differences in CSF cytokine levels among the three groups: NIC, NMOSD AQP4+, and RRMS. A significant difference was observed for several CSF cytokines levels across the groups, indicating distinct cytokine profiles. Specifically, IL-1β (*p* = 0.041), G-CSF (*p* = 0.011), TNF-α (*p* < 0.001), Eotaxin (*p* = 0.008), and MIP-1α (*p* = 0.006) displayed statistically significant differences. To further explore the differences in CSF cytokines levels between the three groups we performed a post-hoc analyses using the Mann-Whitney U test (Fig. 1). The results indicated that IL-1β levels were significantly higher in NMOSD AQP4 + compared to RRMS (*p* = 0.016), while no significant difference was observed between NMOSD AQP4 + and NIC (*p* = 0.068). TNF-α levels displayed a highly significant increase in NMOSD AQP4 + compared to both NIC (*p* < 0.001) and RRMS (*p* = 0.045). For G-CSF, a significant difference was found between NMOSD AQP4 + and NIC (*p* = 0.026), but not between NMOSD AQP4 + and RRMS (*p* = 0.872). Eotaxin levels were significantly different between NMOSD AQP4 + and RRMS (*p* = 0.009), whereas no significant difference was observed between NMOSD AQP4 + and NIC (*p* = 0.216). MIP-1α showed a significant difference between NMOSD AQP4 + and NIC (*p* = 0.015), but not between NMOSD AQP4 + and RRMS (*p* = 0.530) (Fig. 1). No significant associations emerged in the analysis of the other CSF cytokines levels.

### IL-1 CSF concentrations influences NMOSD AQP + disability

**Fig. 2:**
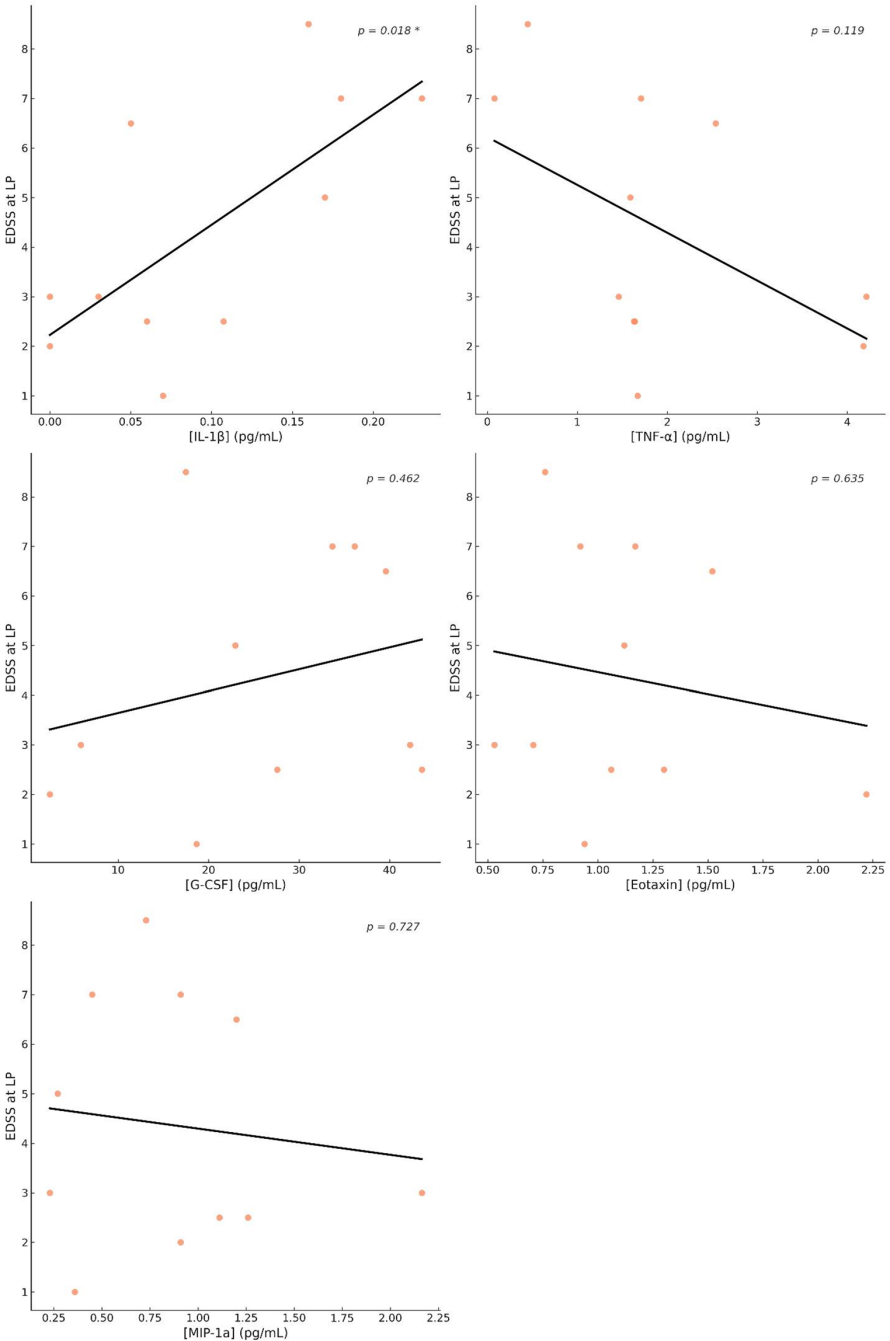
Relationship between EDSS at LP and CSF IL-1β levels in NMOSD AQP4 + patients. Linear regression analyses were conducted to evaluate the relationship between EDSS at LP and IL-1β levels. A significant positive correlation was observed between IL-1β and EDSS at LP (β = 22.24, SE = 7.73, *p* = 0.018, adjusted R² = 0.24), indicating that higher IL-1β levels are associated with increased disability in NMOSD AQP4 + patients. An asterisk (*) denotes statistical significance (*p* < 0.05). Abbreviations: EDSS: Expanded disability status scale; LP: Lumbar Puncture; NMOSD: Neuromyelitis optica spectrum disorder; AQP4: Aquaporin-4; IL: Interleukin; G-CSF: granulocyte-colony stimulating factor; TNF-α: tumor necrosis factor-alpha; MIP-1α: Macrophage Inflammatory Protein-1 alfa; Eotaxin. Missing data: eotaxin: 2 RRMS; MIP-1α: 2 RRMS

A linear regression analysis was conducted to evaluate the relationship between IL-1β levels in CSF and clinical parameters, including disability as measured by the EDSS at the time of LP, age, sex, disease duration, relapse distance. This analysis revealed a significant association between IL-1β and EDSS at LP (B = 22.24, SE = 7.73, *p* = 0.018) (Fig. 2). No significant associations emerged in the analysis of the other CSF cytokines levels.

A further linear regression was performed to determine whether age moderates the relationship between IL-1β and EDSS at LP. In this analysis, both IL-1β (B = 18.96, SE = 6.33, *p* = 0.017) and age (B = 0.09, SE = 0.04, *p* = 0.040) were significantly associated with EDSS at LP.

Additionally, a regression analysis was conducted to assess the potential influence of relapse interval on the relationship between IL-1β and EDSS at LP. IL-1β remained significantly associated with EDSS at LP (B = 18.96, SE = 6.33, *p* = 0.040), whereas relapse interval did not show a significant association (B = 0.09, SE = 0.27, *p* = 0.729).

## Discussion

In this study, we compared CSF cytokine levels among NMOSD AQP4 + patients, RRMS patients, and NIC. We found that NMOSD AQP4 + patients presented elevated inflammatory levels compared to both RRMS patients and NIC, particularly for IL-1β and TNF-α, supporting a heightened innate inflammatory response in NMOSD [9–11]. Moreover, IL-1β CSF levels significantly correlated with EDSS at LP, consistent with its role in amplifying immune responses through neutrophil and leukocyte recruitment [11]. Furthermore, previous studies have shown increased IL-1β synthesis by glial cells during NMOSD relapses [9, 11]. In a preclinical study, IL-1β released in NMOSD AQP4 + lesions, together with IL-1β-induced production and accumulation of complement factors like C1q, was shown to promote neutrophil infiltration and blood-brain barrier disruption around NMOSD AQP4 + lesions. This process may represent a critical secondary factor in lesion formation, facilitating rapid lesion expansion and increased immune cell recruitment to the site [12].

The role of IL-1β is well documented in RRMS [13] where inflammasome activation and IL-1 synthesis is markedly increased during relapses [14]. Interestingly, the study conducted by Blandford et al.l indicates that during remission periods, IL-1β levels in the CSF correlates with greater disability progression over time. The study authors propose that elevated IL-1β levels persisting during phases of low disease activity signify inflammatory mechanisms contributing to progression independent of relapse activity in RRMS patients [15]. In NMOSD AQP4+, the potential relationship between IL-1β levels and disability during the first year of the disease has yet to be fully clarified. However, in our study, we observed a significant association between IL-1β levels in the CSF and disability at the time of LP, suggesting that IL-1β may play a role in neurological impairment even in NMOSD AQP4 + patients. In addition to IL-1β, TNF-α is also a well-known pro-inflammatory cytokine, with established involvement in RRMS [13]. Our findings suggest that TNF-α activity is even more pronounced in NMOSD AQP4 + patients, potentially contributing to more severe lesion formation compared to RRMS [16, 17]. Notably, TNF-α has been linked to astrocyte damage and disruption of the blood-brain barrier (BBB) in NMOSD [17], as well as the induction of neutrophil infiltration across the BBB, which can further exacerbate lesion formation in the spinal cord and optic nerve [17, 18]. No significant differences in G-CSF and MIP-1α levels were observed between NMOSD AQP4 + and RRMS patients; however, both CSF cytokines levels were elevated compared to NIC. These similar levels in NMOSD AQP4 + and RRMS patients may suggest shared mechanisms of immune activation between the conditions. Previous research reported higher CSF concentrations of G-CSF in NMOSD patients compared to RRMS patients during relapse and compared to a control group [19]. While the exact role of G-CSF and MIP-1α (CCL3) in NMOSD remains to be fully elucidated, granulocyte and macrophage involvement has been linked to key pathophysiological features of NMOSD, including complement activation, demyelination, and neuronal loss [20]. In contrast to G-CSF and MIP-1α, we found that eotaxin levels were similar between NMOSD AQP4 + patients and NIC but significantly reduced in RRMS. This finding may indicate a distinct pattern of immune cell trafficking or chemokine activity in NMOSD compared to RRMS patients [10]. Consistent with our findings, elevated levels of peripheral and central eotaxin (CCL11) have been linked to eosinophil recruitment in the perivascular and meningeal spaces of NMOSD lesions during both relapsing [21] and remitting phases [10]. A previous study suggested that chronically elevated levels of TNF-α and IL-1β are closely correlated with eotaxin concentrations and eosinophil activity, potentially contributing to relapses in NMOSD patients during remission [10]. Although previous studies reported increased IL-6 CSF levels in NMOSD patients [22] and some associations with more severe clinical and cognitive deficits [23], we did not observe differences in IL-6 levels between NMOSD AQP4 + patients, RRMS patients, and NIC. Notably, IL-6 is known to play a key role in NMOSD pathogenesis by promoting naive T cell differentiation into pro-inflammatory helper T cells and facilitating B cell transformation into AQP4-IgG-producing plasmablasts, leading to inflammation, blood-brain barrier disruption, and astrocyte damage [24, 25].

An important limitation of our study is the relatively small sample size in both the NMOSD AQP4 + and RRMS groups, which may limit the generalizability of the findings. In particular, the limited number of NMOSD AQP4 + patients may result in reduced statistical power, increasing the risk of type II error.

Regarding disease activity at the time of lumbar puncture, both NMOSD AQP4 + and RRMS groups included a mix of patients with and without gadolinium-enhancing lesions on MRI, reflecting heterogeneity in disease phase (i.e., both active and remission states). Importantly, the proportion of patients with radiological evidence of disease activity was comparable between the two groups, with no statistically significant differences. Nonetheless, this heterogeneity in clinical and MRI activity, combined with the inclusion of NMOSD AQP4 + patients presenting with either optic neuritis or myelitis, may have introduced variability that could affect CSF cytokine profiles and their relationship with neurological disability.

Nonetheless, our findings suggest that NMOSD AQP4 + patients may exhibit a heightened intrathecal inflammatory profile compared to RRMS patients and non-inflammatory controls, particularly with elevated levels of IL-1β and TNF-α. The significant association between IL-1β levels and disability at the time of LP supports its potential role as a biomarker of disease severity in NMOSD, although this relationship should be interpreted with caution given the observational and cross-sectional nature of the study design. Future longitudinal studies with larger and more clinically homogeneous cohorts will be essential to confirm these findings and to explore the prognostic and therapeutic relevance of IL-1β and other inflammatory cytokines in NMOSD.

## Supplementary Information

Below is the link to the electronic supplementary material.

Supplementary file 1 (DOCX 15.9 KB)

## Data Availability

Anonymized datasets are available upon reasonable request to the corresponding author.
