# Supplementary material for: IL-1β contributes to neurological disability in NMOSD AQP4 + Patients
Source: Neurol Sci. 2025 Oct 8;46(12):6687–95. doi: 10.1007/s10072-025-08526-8 (PMC12678549; doi:10.1007/s10072-025-08526-8)
Supplement: Supplementary file 1 [file 10072_2025_8526_MOESM1_ESM.docx]

| Cytokine | β-Coefficient | Standard Error | p-value |
| --- | --- | --- | --- |
| IL-1β | 22.24 | 7.73 | 0.02* |
| IL-2 | 1.72 | 5.16 | 0.75 |
| IL-4 | 1.77 | 4.66 | 0.71 |
| IL-5 | -0.06 | 0.21 | 0.80 |
| IL-6 | 0.07 | 0.10 | 0.49 |
| IL-7 | 0.04 | 0.59 | 0.95 |
| IL-8 | 0.05 | 0.04 | 0.29 |
| IL-10 | 0.24 | 0.70 | 0.74 |
| IL-12 | 3.03 | 2.88 | 0.32 |
| IL-13 | -0.12 | 0.29 | 0.68 |
| IL-17 | 0.86 | 1.18 | 0.48 |
| G-CSF | 0.04 | 0.06 | 0.46 |
| GM-CSF | 13.12 | 25.87 | 0.62 |
| IFN-γ | -0.17 | 0.42 | 0.70 |
| MCP-1 | -0.01 | 0.01 | 0.25 |
| MIP-1β | -0.24 | 0.30 | 0.44 |
| TNF-α | -0.97 | 0.56 | 0.12 |
| Hu IL-1ra | 0.05 | 0.05 | 0.27 |
| IL-9 | 0.22 | 0.23 | 0.36 |
| IL-15 | -0.88 | 0.88 | 0.34 |
| Eotaxin | -0.88 | 1.80 | 0.63 |
| FGF basic | 0.48 | 0.56 | 0.41 |
| IP-10 | -0.0 | 0.00 | 0.46 |
| PDGF | -0.21 | 0.63 | 0.75 |
| RANTES | -0.04 | 0.20 | 0.86 |
| VEGF | 0.02 | 0.03 | 0.51 |
| MIP-1a | -0.53 | 1.47 | 0.73 |

Supplementary Table 1. Linear Regression Analysis of Cytokine Levels and EDSS in NMOSD-AQP4+ Patients. An asterisk (*) denotes statistical significance (p < 0.05). Abbreviations: EDSS: Expanded Disability Status Scale; LP: Lumbar Puncture; IL: Interleukin; TNF-α: Tumor Necrosis Factor-alpha; G-CSF: Granulocyte Colony-Stimulating Factor; GM-CSF: Granulocyte-Macrophage Colony-Stimulating Factor; MCP-1: Monocyte Chemoattractant Protein-1; MIP-1β: Macrophage Inflammatory Protein-1 beta; PDGF: Platelet-Derived Growth Factor; VEGF: Vascular Endothelial Growth Factor. Missing data: eotaxin: 2 RRMS; MIP-1α: 2 RRMS; PDGF: 2 RRMS; VEGF:2RRMS.
